# Prediction and Risk Factors for Prognosis of Cirrhotic Patients with Hepatic Encephalopathy

**DOI:** 10.1155/2021/5623601

**Published:** 2021-10-18

**Authors:** Ying Peng, Qinglin Wei, Yun Liu, Zhenyu Wu, Hongjia Zhang, Hongbo Wu, Jin Chai

**Affiliations:** ^1^Cholestatic Liver Diseases Center and Department of Gastroenterology, The First Affiliated Hospital of Army Medical University, Chongqing, China; ^2^Department of Gastroenterology, The First Affiliated Hospital of Army Medical University, Chongqing, China; ^3^Department of Gastroenterology, The Seventh Medical Center of PLA General Hospital, Beijing 100700, China

## Abstract

**Background and Aims:**

Hepatic encephalopathy (HE) is characterized by recurrence and poor quality of life. Acute-on-chronic liver failure (ACLF) mainly occurs in patients with chronic liver diseases and often presents with HE. Several predictive models have been proposed to predict the outcomes of these patients. Our study is aimed at identifying associated risk factors and the prognostic accuracies of predictive models in HE patients with or without ACLF.

**Methods:**

Patients with liver cirrhosis were retrospectively enrolled. Risk factors were evaluated by multivariate regression analyses. The predictive capabilities of models were calculated using the receiver operating characteristic (ROC) curve analyses and compared by the DeLong tests. Outcomes were defined as in-hospital mortality, HE severity, and ACLF occurrence.

**Results:**

In multivariate regression analyses, serum biomarkers neutrophil and total bilirubin (TBIL) were independently correlated with in-hospital death. Alanine aminotransferase (ALT) and blood urea nitrogen (BUN) were independent serum biomarkers associated with HE severity. Hemoglobin, TBIL, BUN, and international normalized ratio (INR) were significant indicators associated with ACLF incidence. For prediction of in-hospital mortality, Child-Pugh was superior to the others in the whole patients, while NLR showed the best capability in the ACLF group.

**Conclusion:**

In cirrhotic patients present with HE, BUN is a risk factor associated with HE severity and ACLF incidence. Child-Pugh and NLR scores may be effective prognosticators in patients with HE.

## 1. Introduction

Hepatic encephalopathy (HE) is one of the most severe complications of liver cirrhosis, which is also responsible for the major cause of admissions and high mortality in cirrhotic patients. HE has been classified into five grades consisting of progressive stages of mental disorders based on the West Haven criterion. To avoid subjective prejudice, HE is presently classified into two types, covert hepatic encephalopathy (CHE) and overt hepatic encephalopathy (OHE), according to its severity [1]. It has been reported that HE affects more than one-third of cirrhotic patients, of which OHE is irreversible and accounts for more than 30% to 50% of these patients [2].

It has been proven that the occurrence of HE is strongly associated with previous episodic HE in hospitalized cirrhotic patients. Patients manifesting with HE will have a higher risk of progression to acute-on-chronic liver failure (ACLF) and result in poor prognosis in comparison to those without [3]. ACLF, characterized by organ failures and high short-term mortality, will substantially increase the economic burden and medical utilization of patients with chronic liver diseases [4]. To this end, identifying and diagnosing HE patients at an early stage and better prognostication are essential for reducing healthcare burden and mortality.

Various models for monitoring and predicting outcomes in patients with liver diseases have been proposed and validated. However, there is no consensus on which model should be chosen when applying to different populations. Child-Pugh and the model for end-stage liver disease (MELD) score, the well-known prognostic tools of liver function, have been widely used for the prediction of patients with liver diseases. Biggins et al. have conducted a prospective multicenter study enrolling patients with end-stage liver diseases. Originated from the MELD algorithm, they proposed a new score, the model for end-stage liver disease-sodium (MELD-Na), the predictive ability of which was more accurate than that of MELD [5]. The albumin-bilirubin (ALBI) score was initially validated to assess the outcome of patients with hepatocellular carcinoma (HCC), and its effectiveness has been confirmed by relevant studies [6–8]. The neutrophil to lymphocyte ratio (NLR) score, an indicator representing inflammation, has been widely used as a predictive tool for various diseases [9–11].

Few studies have compared the predictive capabilities of the above scores. The previous study explored the prognostic factors correlated with 180 cirrhotic patients presenting with HE who were admitted in the medical intensive care unit (ICU). The researchers found that systolic blood pressure < 90 mmHg, total WBC > 12000 n/mm^3^, and use of mechanical ventilation were significant risk factors for mortality. However, SAPS II, Acute Physiology and Chronic Health Evaluation II (APACHE II), Child-Pugh, and GCS had no significant difference between survivors and nonsurvivors [12]. Therefore, we conduct a retrospective study to investigate the accuracies of Child-Pugh, MELD, MELD-Na, ALBI, and NLR scores in predicting in-hospital mortality of cirrhotic patients with HE with or without ACLF. We also detected the associated risk factors for the severity of HE, and the occurrence of ACLF and in-hospital death.

## 2. Patients and Methods

All patients admitted to the First Affiliated Hospital of Army Medical University from January 2016 to August 2020 were searched through an electronic medical record database. We retrospectively selected patients who were diagnosed with liver cirrhosis and manifested with HE.

The exclusion criteria were as follows: (1) patients with readmissions, (2) patients with HCC or other malignancies, (3) patients with primary neurological diseases or mental disorders, and (4) patients without completed data.

Demographic data, medical history, comorbidities, clinical presentation, laboratory tests, grades of HE, presenting with or without ACLF, and in-hospital mortality were reviewed. HE was classified according to the West Haven criteria. Child-Pugh, MELD, MELD-Na, NLR, and ALBI scores were calculated in all groups. To explore the factors associated with the severity of HE, serum laboratory indicators and noninvasive prognostic models were compared with patients with a low grade (I or II) and high grade (III or IV). HE often occurs in the setting of ACLF and leads to short-term survival; thus, we further detected the characteristics in association with ACLF and in-hospital death. The accuracies of Child-Pugh, MELD, MELD-Na, NLR, and ALBI scores in the prediction of in-hospital death were compared in all the populations and the ACLF patients. The clinical research was authorized by the Ethics Committee Board of Southwest Hospital (KY2020202).

Child-Pugh score calculation consists of total bilirubin, albumin, INR, ascites, and HE. Child-Pugh is classified into A (5-6), B (7-9), and C (10-15) grades [13–15].

(1) $$\begin{array}{c} \text{MELD}\left[14\right]=9.57*\text{loge }\left(\text{creatinine }\left(\text{mg}/\text{dl}\right)+3.78*\text{loge }\left(\text{bilirubin}\right.\;\left(\text{mg}/\text{dl}\right)\right)+11.2*\text{loge }\left(\text{INR}\right)+6.43 \end{array}$$

The creatinine value >4 is set to 4, the minimum values of the three variables is set to 1. The maximum score is limited to 40.

(2) $$\begin{array}{c} \text{MELD}-\text{Na}\left[5\right]=\text{MELD}+1.59*\left(135–\text{Na }\left(\text{mmol}/\text{L}\right)\right) \end{array}$$

The value of serum Na ranges from 120 to 135.

(3) $$\begin{array}{c} \text{ALBI}\left[7\right]=-0.085*\left(\text{albumin }\left(\text{g}/\text{L}\right)+0.66*\log10\left(\text{bilirubin}\right.\;\left(\mu \text{mol}/\text{L}\right)\right)\; \end{array}$$

ALBI score is divided into three grades: ≤−2.6 (grade 1); >−2.6 and ≤ −1.39 (grade 2); >−1.39 (grade 3).

(4) $$\begin{array}{c} \text{NLR }\left[15\right]=\frac{\text{neutrophil count}}{\text{lymphocyte count}}. \end{array}$$

### 2.1. Statistical Analysis

Continuous data were shown as mean ± standard deviation (SD) or median (interquartile range). Categorical data were shown as frequency (percentage). Comparisons between normally distributed continuous data were used by Student's independent *t*-test, while nonnormal distributed data were used by the Mann-Whitney *U* test. Categorical data were compared using the chi-square test or Fisher's exact test. Logistic regression models were used to identify risk factors for HE severity, ACLF incidence, and hospitalized death. Analyses were performed on SPSS version 23.0. The predictive capabilities of scores were calculated using the receiver operating characteristic (ROC) curve analyses. The areas under the ROC curves (AUCs) with 95% confidence intervals (CIs) were compared by the DeLong tests. The cut-off value, sensitivity, specificity, positive likelihood ratio (LR), and negative LR, positive predictive value (PV), and negative PV were also presented. ROC analyses were performed by using MedCalc version 11.4.2.0. A two-sided *p* value < 0.05 was considered significantly different.

## 3. Results

### 3.1. Baseline Characteristics of the Whole Patients

A total of 304 patients were eligible for this study after exclusion. Among the whole patients, 242 patients were male (79.6%). The predominant etiology of liver cirrhosis was HBV infection (65.5%), and the second was alcohol abuse (14.5%). Regrettably, ammonia was only collected in 198 patients. The number of patients presenting with HE grade I/II and grade III/IV was 231 and 73, respectively. The mean Child-Pugh, ALBI, MELD, MELD-Na, and NLR scores were 11.0 ± 2.0, −1.1 ± 0.5, 21.8 ± 8.4, 23.2 ± 8.5, and 6.4 ± 8.0, respectively. In-hospital deaths occurred in 64 patients (21.1%).

### 3.2. Variables Associated with In-Hospital Death

We compared the clinical characteristics between hospitalized survivors and nonsurvivors. Comparative data showed that white blood count (WBC), neutrophil, total bilirubin (TBIL), direct bilirubin (DBIL), indirect bilirubin (IBIL), alanine aminotransferase (ALT), aspartate aminotransferase (AST), blood urea nitrogen (BUN), creatinine, ammonia, prothrombin time (PT), activated partial thromboplastin time (APTT), international normalized ratio (INR), Child-Pugh, ALBI, MELD, MELD-Na, and NLR scores were statistically different between survivors and nonsurvivors (Table 1). The significantly different characteristics between the two groups were included in the multivariate logistic regression models, which were performed to identify independent risk factors. We precluded DBIL, IBIL, PT, APTT, ammonia (106 patients lacked data of ammonia), and five prognostic models to avoid collinearity. Neutrophil and TBIL were found independently correlated with in-hospital mortality (Table S1).

### 3.3. Diagnostic Accuracies of Five Models in the Whole Patients

**Figure 1:**
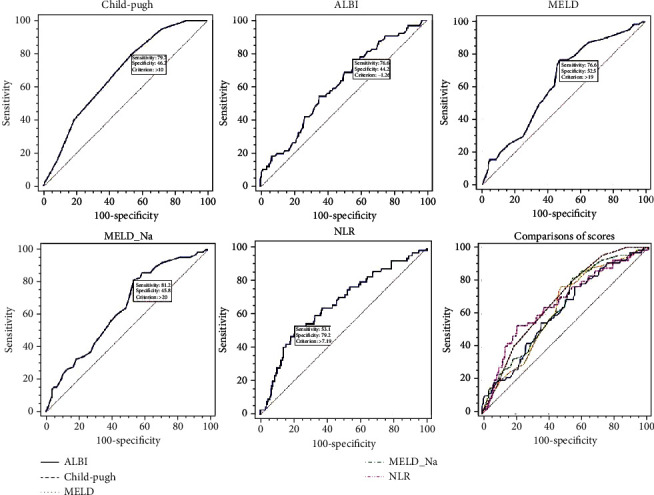
Comparisons of scores in the prediction of in-hospital mortality in the whole patients.

The AUCs of Child-Pugh, ALBI, MELD, MELD-Na, and NLR in the prediction of in-hospital death were 0.681 (95% CI: 0.626-0.733, *p* < 0.0001), 0.615 (95% CI: 0.558-0.670, *p* = 0.003), 0.630 (95% CI: 0.573-0.684, *p* = 0.0005), 0.640 (95% CI: 0.583-0.694, *p* = 0.0002), and 0.664 (95% CI: 0.608-0.717, *p* < 0.0001), respectively (Table 2, Figure 1). The Child-Pugh score showed better predictive performance than the other four models. When compared among these five models, statistical difference was only found between Child-Pugh and ALBI (*p* = 0.031). There were no differences among other comparisons.

### 3.4. Variables Associated with HE Severity

Deaths occurred in 32 of 231 patients in mild (grade I or II) HE and 32 of 76 patients in severe (grade III or IV) HE groups, respectively, which showed significant differences. Gender, age, vital signs, and etiologies of cirrhosis presented no statistically significant differences between the two groups. Blood routine tests including WBC, red blood count (RBC), and neutrophil were significantly different in comparison. As for the serum liver function tests, TBIL, IBIL, ALT, and AST were significantly different. Besides, significant differences were detected in BUN, ammonia, and patients manifesting with ascites between comparisons of the two groups (Table 3). Statistical differences were observed in the Child-Pugh class/score (*p* < 0.001), ALBI grade (*p* = 0.044), MELD score (*p* = 0.043), and NLR scores (*p* = 0.015) between the two groups. In the multivariate logistic regression models, only ALT and BUN were significantly associated with HE severity (Table S2).

### 3.5. Variables Associated with ACLF Incidence

The characteristics of patients with and without ACLF were shown in Table 4. A total of 133 patients suffered from ACLF, and 171 patients were exempted from ACLF. The mortality was 28.9% and 15.2%, respectively. Higher levels of WBC, RBC, hemoglobin, neutrophil, and lymphocyte were observed in patients with ACLF in comparison to those without. The ACLF group also exhibited more severe liver dysfunction (higher levels of liver serological indexes and prognostic scores). Multivariate regression analysis revealed that hemoglobin, TBIL, BUN, and INR were independent variables concerning ACLF occurrence (Table S3).

### 3.6. Diagnostic Accuracies of Five Scores in the ACLF Subgroup

**Figure 2:**
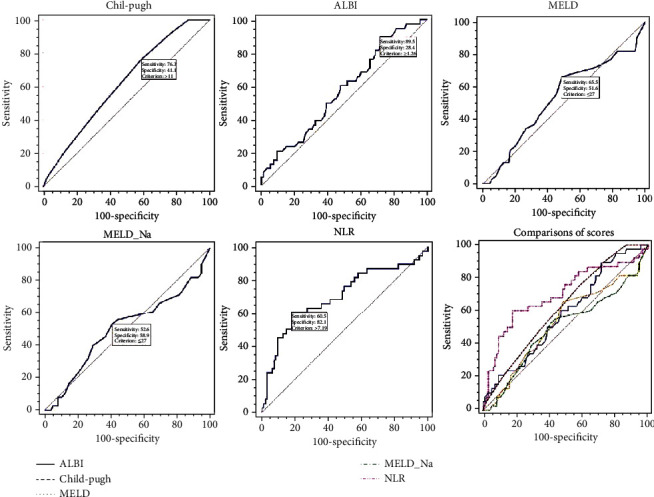
Comparisons of scores in the prediction of in-hospital mortality in the acute-on-chronic liver failure subgroup.

The AUCs of Child-Pugh, ALBI, MELD, MELD-Na, and NLR to predict in-hospital death in the ACLF group were 0.621 (95% CI: 0.533-0.703, *p* = 0.0165), 0.578 (95% CI: 0.489-0.663, *p* = 0.1487), 0.531 (95% CI: 0.443-0.618, *p* = 0.5870), 0.500 (95% CI: 0.412-0.588, *p* = 0.9963), and 0.701 (95% CI: 0.616-0.778, *p* = 0.0003), respectively (Table 2, Figure 2). NLR performed superior discriminative ability to the other four scores in the ACLF subgroup. When compared among these five scores, statistical difference was found between NLR and MELD-Na (*p* = 0.0309). No significant differences were observed among other comparisons.

## 4. Discussion

This retrospective study is aimed at detecting the associated risk factors and selecting suitable prognostic assessment tools of cirrhotic patients presenting with HE. Several findings in our present research need to be addressed.

Firstly, of the whole population, the Child-Pugh score had superior discriminative ability to other scores in assessing in-hospital mortality. It is well known that the Child-Pugh score is widely used as the criterion for the evaluation of liver function in patients with underlying liver diseases in clinical settings. HE grade is one of the indicators that is composed of Child-Pugh calculation, which may contribute to the superiority. This finding is consistent with previous relevant researches. The study conducted by Bhanji et al. revealed that the Child-Pugh class of patients with HE was higher than that of those without [16]. In a prospective study, Duah et al. found that Child-Pugh score elevation was independently associated with the incidence of HE in hospitalized cirrhotic patients [17]. Taş et al. investigated the predictive performances of noninvasive models in cirrhotic patients with HE who were admitted to ICU, followed by chronic liver failure-sequential organ failure assessment (CLIF-SOFA), APACHE II, and Child-Pugh score, which showed a better discriminative value of prognosis than MELD [18]. Patients admitted to ICU were under severe conditions, mostly complicated with organ failures or comorbidities, which might account for the advantages of models evaluating organ failures or serious conditions. Liu et al. led a retrospective study that analyzed cirrhotic patients who suffered from transjugular intrahepatic portosystemic shunt (TIPS). Child-Pugh was identified as an independent risk indicator of the incidence of OHE after TIPS. In this study, a newly established scale incorporating Child-Pugh and spleen volume was proposed as a reliable predictor [19].

Secondly, in our ACLF subgroup, NLR exhibited better predictive accuracy than other scores in predicting hospital death. ACLF is an acute and fatal syndrome that mainly affects patients with preexisting chronic liver diseases. Inflammation is considered one of the precipitating factors and participates in the progression of ACLF, and immune dysfunction is also observed in ACLF patients, which may explain the superiority of NLR; the indicator represents inflammation and immunity. Bernsmeier et al. conducted a multicenter study enrolling cirrhotic patients who developed acute decompensation and ACLF. NLR and monocyte-lymphocyte ratio were independent indicators of in-hospital death [20]. Miao et al. performed a single-center retrospective study to propose that elevated NLR was independently correlated with HBV-related ACLF poor outcome, and its combination with the chronic liver failure-organ failure (CLIF-OF) score could be applied for better prediction of the prognosis of patients [21]. Liu et al. suggested that NLR could be used as a prognostic biomarker in the prediction of 8-week mortality of HBV-related ACLF [22]. A study by Lin et al. also confirmed the effectiveness of NLR for valuing long-term mortality in ACLF populations [23].

Thirdly, serum indicators including WBC, neutrophil, TBIL, ALT, AST, and BUN were observed to be significantly different between comparisons of all groups. In multivariate analyses, neutrophil and TBIL were the independent risk factors in association with in-hospital mortality. BUN was a risk biomarker concerning HE severity and ACLF incidence. The results indicate that regardless of hepatic, renal, and coagulation deterioration, inflammation may play a vital role in the development of HE and ACLF in cirrhotic patients. Recent studies suggest that other than ammonia, inflammation also involves the pathophysiology and progression of HE. Our study strengthens this viewpoint. Moreover, BUN may be a reliable predictor of outcome in these patients.

Fourthly, although the wide application of antiviral medications increased the eradication of hepatitis B virus (HBV) and hepatitis C virus (HCV), HBV infection is still prevailing in cirrhotic patients in our study.

The occurrence and development of HE are highly associated with impairment of liver function, portal hypertension, skeletal muscle, nutrition, and gut microbe. Therefore, sarcopenia, myosteatosis, and fried frailty index have been testified effectively in the prediction of HE [24–26]. The brief antisocial behavior scale (BABS), which consists of bilirubin, albumin, beta-blocker, and statin use, is also involved in the development of OHE [27]. CHE has a higher risk for the progression of OHE; thus, early identification and diagnosis of CHE are important for reducing recurrence and mortality related to HE. Tests for CHE are mainly aimed at evaluating psychology and neurophysiology, which include the psychometric hepatic encephalopathy score (PHES), critical flicker frequency (CFF), animal naming test (ANT), Epworth sleepiness scale (ESS), continuous reaction time (CRT), inhibitory control test (ICT), and electroencephalography [28]. Combined utilization of risk factors and the above evaluation tools may prevent the progression of OHE and improve survival and quality of life for HE patients.

There are some limitations of our study. Firstly, our data are retrospectively gathered that the absence of laboratory indicators may induce bias of certain results. Secondly, ammonia is a serum biomarker prevalent in HE of cirrhosis, whereas it is not commonly detected in our study. Thirdly, none of the patients was diagnosed with nonalcoholic fatty liver diseases. This phenomenon may be due to the fact that our eligible patients are with severely decompensated cirrhosis. Thus liver biopsy, the golden standard of diagnosis, carries a high risk. Admissions of patients to the hospital at an advanced stage may be another reason. Lastly, we could not explore the predictive abilities of models in the assessment of long-term outcomes.

Noninvasive prognostic tools have been investigated by quite a few studies for the assessment of the severity and outcomes of liver diseases and the incidence of liver-related complications. Simple and accurate biomarkers focus on liver dysfunction, malnutrition, and inflammation, and neuropsychiatric indexes should be proposed by well-conducted studies, which might provide long-term information during follow-up and guide clinicians to make prompt and correct strategies for HE patients. More investigators should do some efforts to establish ideally practical prognosticators, which will better stratify the high-risk patients, therefore improving the outcome and diminishing the mortality in clinical practice.

## 5. Conclusions

This present study provides clinical characteristics and related risk factors of cirrhotic patients exhibiting HE with or without ACLF. WBC, neutrophil, BUN, and serum liver function tests are strongly associated with outcomes of HE patients. This study also suggests that the Child-Pugh score could be applied for HE patients in the prediction of in-hospital death. NLR may be an effective model for the assessment of outcomes in patients complicated with ACLF. Furthermore, prospective studies are aimed at establishing new models to predict outcomes in HE patients that should consider BUN a prognostic biomarker.

## Figures and Tables

**Table 1:** Comparative data of survivors versus nonsurvivors.

Variable	Survivors (*n* = 240)			Nonsurvivors (*n* = 64)			*p* value
	No. of patients (*n*)	Mean ± SD or no. (%)	Median (IQR)	No. of patients (*n*)	Mean ± SD or no. (%)	Median (IQR)	
Gender (male, %)	240	189 (78.8)		64	53 (82.8)		0.923
Age (years)	240	52.0 ± 12.1	52.0 (43.0-60.0)	64	52.7 ± 11.2	53.0 (45.0-61.8)	0.668
Vital signs							
Systolic blood pressure (mmHg)	240	117.4 ± 16.7	116.0 (105.0-128.0)	64	118.3 ± 16.7	116.0 (106.3-127.5)	0.701
Diastolic blood pressure (mmHg)	240	69.4 ± 11.0	68.0 (63.0-75.0)	64	71.3 ± 12.5	69.5 (62.0-79.0)	0.251
Heart rate (b.p.m.)	240	87.4 ± 14.7	86.0 (78.0-95.8)	64	85.7 ± 12.8	86.0 (77.0-92.0)	0.390
Etiologies of liver diseases, *n* (%)	240			64			0.424
HBV		153 (63.7)			46 (71.9)		
HCV		5 (2.1)			0 (0)		
Alcohol		37 (15.4)			7 (10.9)		
HBV+HCV		1 (0.4)			0 (0)		
HBV+alcohol		8 (3.3)			3 (4.7)		
HCV+alcohol		3 (1.3)			0 (0)		
DILI		1 (0.4)			0 (0)		
AIH		3 (1.3)			2 (3.1)		
PBC+AIH		2 (0.8)			1 (1.6)		
HBV+AIH		1 (0.4)			0 (0)		
HBV+DILI		2 (0.8)			0 (0)		
Unknown		24 (10.0)			5 (7.8)		
Laboratory tests							
WBC (10^12^/L)	240	6.9 ± 4.5	5.7 (3.9-8.6)	64	9.8 ± 6.4	8.8 (5.8-12.6)	<0.001^∗^
RBC (10^12^/L)	240	3.1 ± 0.9	3.0 (2.5-3.8)	64	3.1 ± 1.1	3.1 (2.2-3.9)	0.748
Hemoglobin (g/L)	239	100.5 ± 26.9	97.0 (80.0-118.0)	64	101.2 ± 30.9	100.0 (80.0-123.5)	0.864
Platelet (10^9^/L)	239	82.1 ± 54.1	66.0 (47.0-105.0)	64	81.1 ± 53.9	63.5 (40.0-123.5)	0.611
Neutrophil (10^12^/L)	240	4.9 ± 3.7	3.6 (2.4-6.4)	64	7.8 ± 6.0	6.4 (4.2-9.9)	<0.001^∗^
Lymphocyte (10^12^/L)	240	1.2 ± 1.0	0.9 (0.6-1.5)	64	1.3 ± 1.3	1.0 (0.7-1.4)	0.818
TBIL (*μ*mol/L)	240	197.7 ± 190.2	102.4 (43.3-349.3)	64	332.8 ± 220.3	319.0 (139.9-510.1)	<0.001^∗^
DBIL (*μ*mol/L)	240	113.0 ± 118.1	50.8 (16.5-203.7)	64	195.3 ± 138.7	179.8 (71.8-285.6)	<0.001^∗^
IBIL (*μ*mol/L)	240	80.4 ± 78.5	46.8 (22.5-110.7)	64	141.9 ± 98.9	132.3 (51.4-211.5)	<0.001^∗^
Albumin (g/L)	240	29.8 ± 5.1	29.7 (26.4-33.5)	64	29.6 ± 5.0	30.3 (25.5-32.5)	0.748
ALT (U/L)	240	186.3 ± 427.6	39.5 (24.5-105.3)	64	292.9 ± 477.2	82.6 (38.6-305.3)	0.001^∗^
AST (U/L)	240	202.2 ± 423.7	65.4 (39.1-155.1)	55	359.2 ± 471.0	130.5 (65.4-360.1)	<0.001^∗^
ALP (U/L)	230	145.4 ± 81.3	123.5 (97.0-170.8)	64	131.0 ± 67.7	119.0 (80.3-181.3)	0.250
GGT (U/L)	240	87.6 ± 113.7	54.0 (30.2-99.0)	64	102.9 ± 100.6	68.5 (41.3-128.8)	0.076
Blood urea nitrogen (mmol/L)	240	8.2 ± 6.2	6.6 (4.4-9.7)	64	11.0 ± 8.1	9.2 (4.9-14.3)	0.005^∗^
Creatinine (*μ*mol/L)	240	92.2 ± 79.9	69.1 (55.0-96.2)	64	112.0 ± 94.0	87.3 (60.6-135.7)	0.012^∗^
Potassium (mmol/L)	240	3.9 ± 0.7	3.9 (3.4-4.3)	63	4.0 ± 0.8	4.0 (3.6-4.6)	0.370
Sodium (mmol/L)	240	136.3 ± 6.6	137.0 (132.0-140.4)	64	134.5 ± 7.8	135.3 (129.3-140.8)	0.061
Calcium (mmol/L)	231	2.2 ± 0.2	2.2 (2.1-2.3)	64	2.2 ± 0.3	2.2 (2.0-2.4)	0.477
Ammonia (*μ*mol/L)	162	59.8 ± 51.7	42.0 (27.8-79.3)	36	87.2 ± 73.9	66.5 (28.8-116.3)	0.031^∗^
PT (second)	240	21.9 ± 9.5	18.7 (15.4-25.6)	64	27.2 ± 11.3	24.6 (18.1-34.7)	<0.001^∗^
APTT (second)	240	55.5 ± 21.4	51.3 (38.7-69.3)	64	65.6 ± 25.8	59.6 (48.8-83.7)	0.004^∗^
INR	240	1.9 ± 0.9	1.6 (1.3-2.2)	64	2.3 ± 1.0	2.2 (1.6-2.8)	<0.001^∗^
Ascites (no/mild/moderate-severe)	240	52/104/84		64	8/34/22		0.066
Hepatic encephalopathy (grades I-II/grades III-IV)	240	199/41		64	32/32		0.545
Child-Pugh score	240	10.7 ± 2.0	11.0 (9.0-12.0)	64	12.0 ± 1.5	12.0 (11.0-13.0)	<0.001^∗^
Child-Pugh class (A/B/C)	240	5/61/174		64	0/3/61		0.958
ALBI score	240	−1.2 ± 0.5	-1.1 (-1.6- (-0.8))	64	−0.9 ± 0.5	-0.9 (-1.2- (-0.7))	0.002^∗^
ALBI grade (1/2/3)	240	1/86/153		64	0/13/51		0.557
MELD score	240	20.9 ± 8.3	19.0 (14.0-27.8)	64	24.8 ± 8.2	24.5 (20.0-29.8)	0.001^∗^
MELD-Na score	240	22.3 ± 8.4	22.0 (15.0-29.0)	64	26.5 ± 8.0	26.0 (21.0-33.0)	0.001^∗^
NLR	240	5.5 ± 5.5	3.7 (2.3-6.6)	64	9.7 ± 13.3	7.4 (3.4-11.1)	<0.001^∗^

Abbreviations: AIH: autoimmune hepatitis; ALBI: albumin to bilirubin; ALP: alkaline phosphatase; ALT: alanine aminotransferase; APTT: activated partial thromboplastin time; AST: aspartate aminotransferase; DBIL: direct bilirubin; DILI: drug-induced liver injury; GGT: gamma-glutamyl transpeptidase; HBV: hepatitis B virus; HCV: hepatitis C virus; IBIL: indirect bilirubin; IQR: interquartile range; INR: international normalized ratio; MELD: model for end-stage liver diseases; MELD-Na: model for end-stage liver diseases-sodium; NLR: neutrophil to lymphocyte ratio; PBC: primary biliary cholangitis; PT: prothrombin time; RBC: red blood count; SD: standard deviation; TBIL: total bilirubin; WBC: white blood count. Note: ^∗^*p* value < 0.05.

**Table 2:** Diagnostic accuracies of Child-Pugh, ALBI, MELD, MELD-Na, and NLR scores.

Prognostic model	Area under the ROC curve	Criterion value	Sensitivity	Specificity	Positive LR	Negative LR	Positive PV	Negative PV	*p* value
The whole patients									
Child-Pugh	0.681 (95% CI: 0.626-0.733)	10.0	79.7	46.3	1.5	0.4	28.3	89.5	<0.0001^∗^
ALBI	0.615 (95% CI: 0.558-0.670)	-1.3	76.6	44.2	1.4	0.5	26.8	87.6	0.0030^∗^
MELD	0.630 (95% CI: 0.573-0.684)	19.0	76.6	52.5	1.6	0.5	30.1	89.4	0.0005^∗^
MELD-Na	0.640 (95% CI: 0.583-0.694)	20.0	81.3	45.8	1.5	0.4	28.6	90.2	0.0002^∗^
NLR	0.664 (95% CI: 0.608-0.717)	7.2	53.1	79.2	2.6	0.6	40.5	86.4	<0.0001^∗^
ACLF subgroup									
Child-Pugh	0.621 (95% CI: 0.533-0.703)	11.0	76.3	41.1	1.3	0.6	34.1	81.2	0.0165^∗^
ALBI	0.578 (95% CI: 0.489-0.663)	-1.3	89.5	28.4	1.3	0.4	33.3	87.1	0.1487
MELD	0.531 (95% CI: 0.443-0.618)	27.0	65.8	51.6	1.4	0.7	35.2	79.0	0.5870
MELD-Na	0.500 (95% CI: 0.412-0.588)	27.0	52.6	59.0	1.3	0.8	33.9	75.7	0.9963
NLR	0.701 (95% CI: 0.616-0.778)	7.2	60.5	82.1	3.4	0.5	57.5	83.9	0.0003^∗^

Abbreviations: ACLF: acute-on-chronic liver failure; ALBI: albumin to bilirubin; CI: confidence interval; HE: hepatic encephalopathy; LR: likelihood ratio; MELD: model for end-stage liver diseases; MELD-Na: model for end-stage liver diseases-sodium; NLR: neutrophil to lymphocyte ratio; PV: predictive value; ROC: receiver operating characteristic. Note: ^∗^*p* value < 0.05.

**Table 3:** Comparative data of patients with mild hepatic encephalopathy versus severe hepatic encephalopathy.

Variable	HE grade I or II (*n* = 231)			HE grade III or IV (*n* = 73)			*p* value
	No. of patients (*n*)	Mean ± SD or no. (%)	Median (IQR)	No. of patients (*n*)	Mean ± SD or no. (%)	Median (IQR)	
Gender (male, %)	231	182 (78.8)		73	60 (82.2)		0.530
Age (years)	231	52.4 ± 11.4	52.0 (44.0-60.0)	73	51.6 ± 13.7	53.0 (40.5-62.0)	0.654
Vital signs							
Systolic blood pressure (mmHg)	231	117.1 ± 16.7	116.0 (105.0-127.0)	73	119.3 ± 16.5	117.0 (104.5-133.0)	0.310
Diastolic blood pressure (mmHg)	231	69.7 ± 11.3	68.0 (63.0-75.0)	73	70.1 ± 11.5	67.0 (61.5-80.0)	0.828
Heart rate (b.p.m.)	231	86.4 ± 14.0	85.0 (78.0-93.0)	73	89.1 ± 15.1	88.0 (78.5-99.0)	0.168
Etiologies of liver diseases	231			73			0.585
HBV		153 (66.2)			46 (63.0)		
HCV		4 (1.7)			1 (1.4)		
Alcohol		30 (13.0)			14 (19.2)		
HBV+HCV		1 (0.4)			0 (0)		
HBV+alcohol		7 (3.0)			4 (5.5)		
HCV+alcohol		3 (1.3)			0 (0)		
DILI		1 (0.4)			0 (0)		
AIH		2 (0.9)			3 (4.1)		
PBC+AIH		3 (1.3)			0 (0)		
HBV+AIH		1 (0.4)			0 (0)		
HBV+DILI		2 (0.9)			0 (0)		
Unknown		24 (10.4)			5 (6.8)		
Laboratory tests							
WBC (10^12^/L)	231	7.2 ± 5.1	5.8 (3.9-9.0)	73	8.5 ± 4.9	7.5 (4.9-10.3)	0.008^∗^
RBC (10^12^/L)	231	3.1 ± 0.9	3.0 (2.5-3.6)	73	3.3 ± 1.0	3.2 (2.6-4.1)	0.034^∗^
Hemoglobin (g/L)	230	99.6 ± 26.2	97.0 (80.0-117.0)	73	104.1 ± 32.0	102.0 (80.0-131.0)	0.271
Platelet (10^9^/L)	230	81.7 ± 53.3	66.0 (46.8-106.3)	73	82.7 ± 56.5	64.0 (40.0-122.0)	0.730
Neutrophil (10^12^/L)	231	5.2 ± 4.5	3.7 (2.4-6.6)	73	6.4 ± 4.0	5.7 (3.1-8.8)	0.004^∗^
Lymphocyte (10^12^/L)	231	1.2 ± 1.1	0.9 (0.7-1.5)	73	1.2 ± 0.9	1.0 (0.6-1.5)	0.853
TBIL (*μ*mol/L)	231	211.9 ± 197.9	123.5 (44.0-371.5)	73	270.9 ± 218.1	260.0 (67.3-421.8)	0.028^∗^
DBIL (*μ*mol/L)	231	123.8 ± 125.9	69.3 (18.3-231.3)	73	151.1 ± 129.2	146.0 (30.1-236.8)	0.055
IBIL (*μ*mol/L)	231	85.1 ± 78.1	51.6 (22.9-137.0)	73	119.6 ± 106.0	93.0 (34.1-180.8)	0.015^∗^
Albumin (g/L)	231	29.7 ± 4.9	29.8 (26.4-32.5)	73	30.0 ± 5.5	29.7 (25.8-34.1)	0.705
ALT (U/L)	231	176.8 ± 397.7	42.1 (23.9-100.6)	73	309.9 ± 543.4	80.3 (33.8-365.8)	0.002^∗^
AST (U/L)	218	199.9 ± 406.8	68.6 (40.9-157.0)	67	333.2 ± 511.6	112.1 (49.3-338.8)	0.015^∗^
ALP (U/L)	231	143.7 ± 79.7	123.0 (95.0-171.0)	73	138.0 ± 76.2	120.0 (80.5-179.5)	0.589
GGT (U/L)	231	85.3 ± 95.9	57.0 (32.0-102.0)	73	108.3 ± 148.8	56.0 (30.0-132.0)	0.569
Blood urea nitrogen (mmol/L)	231	8.3 ± 5.9	6.6 (4.4-9.8)	73	10.4 ± 8.7	8.2 (4.9-12.4)	0.035^∗^
Creatinine (*μ*mol/L)	231	93.3 ± 81.5	70.0 (55.0-100.2)	73	105.9 ± 88.4	74.7 (59.5-122.0)	0.245
Potassium (mmol/L)	223	3.9 ± 0.7	3.9 (3.4-4.3)	73	4.1 ± 0.8	4.0 (3.5-4.7)	0.052
Sodium (mmol/L)	231	136.0 ± 6.6	136.0 (131.7-140.0)	73	135.6 ± 7.8	137.0 (130.6-141.0)	0.652
Calcium (mmol/L)	223	2.2 ± 0.2	2.2 (2.1-2.3)	73	2.1 ± 0.6	2.2 (2.0-2.3)	0.364
Ammonia (*μ*mol/L)	145	56.3 ± 46.5	41.0 (28.0-75.5)	53	87.9 ± 75.2	67.0 (26.5-119.0)	0.012^∗^
PT (second)	231	22.4 ± 9.7	19.0 (15.5-26.8)	73	24.8 ± 11.1	21.3 (16.6-30.5)	0.070
APTT (second)	231	57.4 ± 23.3	52.3 (41.0-70.8)	73	58.6 ± 21.0	56.7 (43.3-72.8)	0.692
INR	231	1.9 ± 0.9	1.6 (1.3-2.3)	73	2.2 ± 1.0	1.9 (1.4-2.6)	0.061
Ascites (no/mild/moderate-severe)	231	50/109/72		73	10/29/34		0.015^∗^
Child-Pugh score	231	10.6 ± 1.9	11.0 (9.0-12.0)	73	12.2 ± 1.7	12.0 (11.0-14.0)	<0.001^∗^
Child-Pugh class (A/B/C)	231	5/59/167		73	0/5/68		<0.001^∗^
ALBI score	231	−1.2 ± 0.5	-1.1 (-1.5- (-0.7))	73	−1.1 ± 0.5	-1.1 (-1.4- (-0.7))	0.338
ALBI grade (1/2/3)	231	1/82/148		73	0/17/56		0.044^∗^
MELD score	231	21.2 ± 8.4	19.0 (14.0-27.0)	73	22.1 ± 9.9	21.5 (14.0-27.8)	0.043^∗^
MELD-Na score	231	22.7 ± 8.5	22.0 (16.0-29.0)	73	23.4 ± 8.3	23.0 (16.0-29.0)	0.069
NLR	231	6.0 ± 7.9	3.9 (2.4-7.0)	73	24.8 ± 8.4	25.0 (18.0-32.0)	0.015^∗^
In-hospital mortality	231	32 (13.9)		73	32 (43.8)		<0.001^∗^

Abbreviations: AIH: autoimmune hepatitis; ALBI: albumin to bilirubin; ALP: alkaline phosphatase; ALT: alanine aminotransferase; APTT: activated partial thromboplastin time; AST: aspartate aminotransferase; DBIL: direct bilirubin; DILI: drug-induced liver injury; GGT: gamma-glutamyl transpeptidase; HBV: hepatitis B virus; HCV: hepatitis C virus; HE: hepatic encephalopathy; IBIL: indirect bilirubin; IQR: interquartile range; INR: international normalized ratio; MELD: model for end-stage liver diseases; MELD-Na: model for end-stage liver diseases-sodium; NLR: neutrophil to lymphocyte ratio; PBC: primary biliary cholangitis; PT: prothrombin time; RBC: red blood count; SD: standard deviation; TBIL: total bilirubin; WBC: white blood count. Note: ^∗^*p* value < 0.05.

**Table 4:** Comparative data of patients with acute-on-chronic liver failure versus without acute-on-chronic liver failure.

Variable	Patients with ACLF (*n* = 133)			Patients without ACLF (*n* = 171)			*p* value
	No. of patients (*n*)	Mean ± SD or no. (%)	Median (IQR)	No. of patients (*n*)	Mean ± SD or no. (%)	Median (IQR)	
Gender (male, %)	133	112 (84.2)		171	130 (76.0)		0.953
Age (years)	133	49.8 ± 11.5	50.0 (41.0-56.0)	171	54.0 ± 11.9	53.0 (45.0-62.0)	0.002^∗^
Vital signs							
Systolic blood pressure (mmHg)	133	117.5 ± 15.5	116.0 (105.5-127.0)	171	117.7 ± 17.5	115.0 (105.0-129.0)	0.954
Diastolic blood pressure (mmHg)	133	70.6 ± 11.1	68.0 (63.0-79.0)	171	69.2 ± 11.5	68.0 (61.0-75.0)	0.296
Heart rate (b.p.m.)	133	87.1 ± 12.8	86.0 (78.0-92.5)	171	87.0 ± 15.4	86.0 (77.0-96.0)	0.621
Etiologies of liver diseases, *n* (%)	133			171			0.061
HBV		116 (87.2)			83 (48.5)		
HCV		0 (0)			5 (2.9)		
Alcohol		5 (3.8)			39 (22.8)		
HBV+HCV		0 (0)			1 (0.6)		
HBV+alcohol		6 (4.5)			5 (2.9)		
HCV+alcohol		0 (0)			3 (1.8)		
DILI		0 (0)			1 (0.6)		
AIH		2 (1.5)			3 (1.8)		
PBC+AIH		1 (0.8)			2 (1.2)		
HBV+AIH		1 (0.8)			0 (0)		
HBV+DILI		0 (0)			2 (1.2)		
Unknown		2 (1.5)			27 (15.8)		
Laboratory tests							
WBC (10^12^/L)	133	8.5 ± 5.6	7.5 (5.2-10.1)	171	6.7 ± 4.5	5.3 (3.6-8.6)	<0.001^∗^
RBC (10^12^/L)	133	3.4 ± 1.0	3.2 (2.7-4.1)	171	3.0 ± 0.8	2.8 (2.4-3.4)	<0.001^∗^
Hemoglobin (g/L)	133	110.2 ± 27.0	108.0 (92.0-133.0)	170	93.3 ± 26.0	90.0 (75.0-109.0)	<0.001^∗^
Platelet (10^9^/L)	133	81.5 ± 49.0	66.0 (46.5-113.5)	170	82.2 ± 57.7	66.0 (44.8-105.0)	0.747
Neutrophil (10^12^/L)	133	6.4 ± 5.1	5.4 (3.3-8.1)	171	4.8 ± 3.7	3.4 (2.2-6.3)	<0.001^∗^
Lymphocyte (10^12^/L)	133	1.3 ± 1.1	1.0 (0.8-1.6)	171	1.1 ± 1.1	0.8 (0.5-1.4)	0.001^∗^
TBIL (*μ*mol/L)	133	360.3 ± 187.6	360.6 (222.8-507.8)	171	121.8 ± 148.0	58.5 (35.6-126.8)	<0.001^∗^
DBIL (*μ*mol/L)	133	251.1 ± 116.6	216.1 (124.0-300.3)	170	64.8 ± 90.6	24.8 (12.5-70.5)	<0.001^∗^
IBIL (*μ*mol/L)	133	140.1 ± 88.3	121.8 (75.6-197.4)	170	57.3 ± 65.8	33.1 (19.8-62.7)	<0.001^∗^
Albumin (g/L)	133	30.4 ± 4.9	30.1 (27.2-33.5)	171	29.3 ± 5.1	29.6 (25.8-32.6)	0.075
ALT (U/L)	133	320.1 ± 515.4	95.0 (49.6-330.7)	171	122.1 ± 348.3	30.1 (22.2-61.7)	<0.001^∗^
AST (U/L)	124	332.4 ± 517.1	136.4 (75.7-347.7)	161	153.4 ± 344.2	50.0 (36.0-89.4)	<0.001^∗^
ALP (U/L)	133	144.4 ± 70.2	123.0 (101.0-166.0)	171	140.7 ± 85.0	121.0 (87.0-176.0)	0.279
GGT (U/L)	133	91.5 ± 85.7	67.0 (40.5-107.9)	171	90.4 ± 127.6	49.0 (23.0-102.0)	0.001^∗^
Blood urea nitrogen (mmol/L)	133	7.9 ± 5.7	6.1 (4.0-9.7)	171	9.5 ± 7.4	7.0 (4.8-11.9)	0.011^∗^
Creatinine (*μ*mol/L)	133	91.4 ± 64.0	68.8 (54.0-97.2)	171	100.2 ± 95.6	71.3 (58.1-102.0)	0.302
Potassium (mmol/L)	133	4.0 ± 0.7	4.0 (3.5-4.4)	170	4.0 ± 0.7	3.9 (3.4-4.4)	0.960
Sodium (mmol/L)	133	134.9 ± 6.8	136.0 (130.4-139.9)	171	136.6 ± 6.9	137.0 (132.5-141.0)	0.028^∗^
Calcium (mmol/L)	128	2.2 ± 0.3	2.2 (2.1-2.4)	166	2.2 ± 0.3	2.1 (2.0-2.3)	0.158
Ammonia (*μ*mol/L)	79	69.8 ± 65.7	46.0 (28.0-92.0)	119	61.4 ± 50.8	46.0 (28.0-81.0)	0.799
PT (second)	133	28.6 ± 10.4	26.4 (21.5-34.8)	171	18.7 ± 7.4	16.6 (14.7-19.5)	<0.001^∗^
APTT (second)	133	68.6 ± 21.1	64.0 (52.3-82.7)	171	49.2 ± 20.2	45.4 (34.1-56.7)	<0.001^∗^
INR	133	2.5 ± 0.9	2.2 (1.8-3.0)	171	1.6 ± 0.6	1.4 (1.3-1.7)	<0.001^∗^
Ascites (no/mild/moderate-severe)	133	15/70/48		171	45/68/58		0.569
Hepatic encephalopathy (grades I-II/grades III-IV)	133	98/35		171	133/38		0.635
Child-Pugh score	133	11.9 ± 1.6	12.0 (11.0-13.0)	171	10.3 ± 1.9	10.0 (9.0-12.0)	<0.001^∗^
Child-Pugh class (A/B/C)	133	1/11/121		171	4/53/114		0.209
ALBI score	133	−1.0 ± 0.5	-0.9 (-1.2- (-0.7))	171	−1.3 ± 0.5	-1.3 (-1.7- (-0.9))	<0.001^∗^
ALBI grade (1/2/3)	133	1/21/111		171	0/78/93		0.694
MELD score	133	26.9 ± 6.7	27.0 (23.0-31.0)	171	17.7 ± 7.3	16.0 (13.0-21.0)	<0.001^∗^
MELD-Na score	133	28.2 ± 6.7	29.0 (24.0-33.0)	171	19.3 ± 7.7	18.0 (14.0-23.0)	<0.001^∗^
NLR	133	6.6 ± 8.9	4.7 (2.5-8.0)	171	6.2 ± 7.2	3.7 (2.4-7.9)	0.182
In-hospital mortality	133	38 (28.6)		171	26 (15.2)		0.878

Abbreviations: ACLF: acute-on-chronic liver failure; AIH: autoimmune hepatitis; ALBI: albumin to bilirubin; ALP: alkaline phosphatase; ALT: alanine aminotransferase; APTT: activated partial thromboplastin time; AST: aspartate aminotransferase; DBIL: direct bilirubin; DILI: drug-induced liver injury; GGT: gamma-glutamyl transpeptidase; HBV: hepatitis B virus; HCV: hepatitis C virus; IBIL: indirect bilirubin; IQR: interquartile range; INR: international normalized ratio; MELD: model for end-stage liver diseases; MELD-Na: model for end-stage liver diseases-sodium; NLR: neutrophil to lymphocyte ratio; PBC: primary biliary cholangitis; PT: prothrombin time; RBC: red blood count; SD: standard deviation; TBIL: total bilirubin; WBC: white blood count. Note: ^∗^*p* value < 0.05.

## Data Availability

We retrospectively collected medical records of patients admitted to our hospital. Our underlying data are not freely available for the ethical policies of our hospital.

## Abbreviations

HE: Hepatic encephalopathy
CHE: Covert hepatic encephalopathy
OHE: Overt hepatic encephalopathy
ACLF: Acute-on-chronic liver failure
MELD: The model for end-stage liver disease
MELD-Na: The model for end-stage liver disease-sodium
ALBI: Albumin-bilirubin
HCC: Hepatocellular carcinoma
NLR: Neutrophil to lymphocyte ratio
ICU: Intensive care unit
APACHE II: Acute Physiology and Chronic Health Evaluation II
SD: Standard deviation
ROC: The receiver operating characteristic
AUC: The areas under the ROC curve
CI: Confidence interval
LR: Likelihood ratio
PV: Predictive value
WBC: White blood count
TBIL: Total bilirubin
DBIL: Direct bilirubin,
IBIL: Indirect bilirubin
ALT: Alanine aminotransferase
AST: Aspartate aminotransferase
BUN: Blood urea nitrogen
PT: Prothrombin time
APTT: Activated partial thromboplastin time
INR: International normalized ratio
CLIF-SOFA: Chronic liver failure-sequential organ failure assessment
TIPS: Transjugular intrahepatic portosystemic shunt
CLIF-OF: The chronic liver failure-organ failure
HBV: Hepatitis B virus
HCV: Hepatitis C virus
BABS: Brief antisocial behavior scale
PHES: Psychometric hepatic encephalopathy score
CFF: Critical flicker frequency
ANT: Animal naming test
ESS: Epworth sleepiness scale
CRT: Continuous reaction time
ICT: Inhibitory control test.

## Supplementary Materials

Supplementary 1 Table S1 Logistic rfegression analysis of risk factors for in-hospital death.

Supplementary 2 Table S2 Logistic regression analysis of risk factors for hepatic encephalopathy severity.

Supplementary 3 Table S3 Logistic regression analysis of risk factors for acute-on-chronic liver failure.
