# Supplementary material for: Prediction and Risk Factors for Prognosis of Cirrhotic Patients with Hepatic Encephalopathy
Source: Gastroenterol Res Pract. 2021 Oct 18;2021:5623601. doi: 10.1155/2021/5623601 (PMC8546404; doi:10.1155/2021/5623601)
Supplement: Supplementary 1 — Table S1 Logistic rfegression analysis of risk factors for in-hospital death. [file 5623601.f1.docx]

| **Table S1. Logistic regression analysis of risk factors of in-hospital death** | | |
| --- | --- | --- |
| **Variables** | **Odds ratio (95%CI)** | **p value** |
| Neutrophil | 1.076 (1.001-1.155) | 0.046 |
| TBIL | 1.003 (1.001-1.004) | 0.001 |
| **Abbreviations:** CI, confidence interval; TBIL, total bilirubin. | | |
