# Supplementary material for: Prediction and Risk Factors for Prognosis of Cirrhotic Patients with Hepatic Encephalopathy
Source: Gastroenterol Res Pract. 2021 Oct 18;2021:5623601. doi: 10.1155/2021/5623601 (PMC8546404; doi:10.1155/2021/5623601)
Supplement: Supplementary 2 — Table S2 Logistic regression analysis of risk factors for hepatic encephalopathy severity. [file 5623601.f2.docx]

| **Table S2. Logistic regression analysis of risk factors of hepatic encephalopathy severity** | | |
| --- | --- | --- |
| **Variables** | **Odds ratio (95%CI)** | **p value** |
| ALT | 1.001 (1.000-1.001) | 0.020 |
| Blood urea nitrogen | 1.047 (1.008-1.089) | 0.019 |
| **Abbreviations:** ALT, alanine aminotransferase | | |
