# Supplementary material for: Prediction and Risk Factors for Prognosis of Cirrhotic Patients with Hepatic Encephalopathy
Source: Gastroenterol Res Pract. 2021 Oct 18;2021:5623601. doi: 10.1155/2021/5623601 (PMC8546404; doi:10.1155/2021/5623601)
Supplement: Supplementary 3 — Table S3 Logistic regression analysis of risk factors for acute-on-chronic liver failure. [file 5623601.f3.docx]

| **Table S3. Logistic regression analysis of risk factors of acute-on-chronic liver failure** | | |
| --- | --- | --- |
| **Variables** | **Odds ratio (95%CI)** | **p value** |
| Hemoglobin (g/L) | 1.013 (1.000-1.026) | 0.044 |
| TBIL (umol/L) | 1.006 (1.004-1.009) | <0.001 |
| Blood urea nitrogen (mmol/L) | 0.915 (0.861-0.973) | 0.005 |
| INR | 2.217 (1.338-3.674) | 0.002 |
| **Abbreviations:** CI, confidence interval; INR, international normalized ratio; TBIL, total bilirubin; | | |
